# The inclusion of black cumin meal improves the carcass characteristics of growing Awassi lambs

**DOI:** 10.14202/vetworld.2021.237-241

**Published:** 2021-01-26

**Authors:** Belal S. Obeidat

**Affiliations:** Department of Animal Production, Faculty of Agriculture, Jordan University of Science and Technology, Irbid 22110, Jordan

**Keywords:** Awassi lambs, black cumin meal, carcass characteristics and meat quality

## Abstract

**Background and Aim:**

Black cumin meal (BCM) is the principal by-product that can be obtained from black cumin seed (*Nigella sativa* L) after the oil is removed from the fruit and represents 70-75% of the fruit weight. The aim of this experiment was to assess the influence of feeding BCM on the carcass characteristics and meat quality of growing lambs.

**Materials and Methods:**

Twenty-four Awassi lambs were randomly divided into two dietary treatments. The lambs were penned individually in single pens and provided access to *ad libitum* feed and water. The dietary treatments were no BCM (control [CON]; n=12) or 150 g BCM/kg (BCM 150; n=12) of dietary dry matter.

**Results:**

Lambs fed BCM had greater (p≤0.01) fasting live BW, and hot and cold carcass weights. Leg fat and eye muscle depth were lower (p≤0.05) in CON versus BCM lambs. Meat whiteness (L*), redness (a*), and yellowness did not differ between lambs fed the two diets.

**Conclusion:**

The inclusion of BCM improves lamb carcass characteristics, but does not affect lamb meat quality.

## Introduction

In Jordan, the sheep population is approximately 3.1 million [1], of which Awassi is the predominant breed [2]. The main purpose of raising sheep in Jordan is for meat production; sheep account for approximately 40% of all red meat consumption [1]. In the Middle East, including Jordan, sheep producers face a number of problems that may constrain feed availability, such as a harsh environment, poor quality forage, and the high cost of feed ingredients, particularly ingredients considered to be a protein source for animals.

Feed ingredients, such as barley grains and soybean meal, tend to increase the cost of animal production due to price fluctuations and the importation process. As such, replacing traditional feed ingredients with alternative feeds may lower production costs and enhance product quality [3]. Feeds derived from plant by-products are considered to be an economical alternative feedstuff for use in animal rations and are reported to enhance performance and carcass traits [4]; among these by-products are black cumin meal (BCM). BCM is the principal by-product derived from black cumin seed (*Nigella sativa* L). It is obtained after the oil is removed from the fruit and represents 70-75% of the fruit weight [5]. The processing of black cumin seed results in a large amount of waste, called BCM, which can possibly be used as supplemental feed for animals [6]. Black cumin (*N. sativa* L) is an annual herb that is widely used in both human and animal medicine. Moreover, it is considered to be a source of industrial seed oil that is usually cropped for seed production [7]. BCM obtained after oil extraction from the seed is reported to contain natural products and secondary metabolites, and it is rich in nutrients such as crude protein (CP), essential amino acids, and essential fats, making it an excellent alternative feed for ruminants [6,8]. Furthermore, Obeidat [6] reported that the inclusion of 15% BCM improved the growth performance and feed intake of Awassi lambs. Similarly, Cherif *et al*. [7] observed that the addition of 12% *N. sativa* seeds to a balanced forage to concentrate ration had a positive effect on the hot and cold carcass yields, and tail weights of Barbarine lambs, compared with a control (CON) diet.

Previous studies have shown the impact of BCM on animal performance [6,7], but few have explained its effect on carcass traits and meat quality. Therefore, the aim of this experiment was to assess the influence of feeding BCM on the carcass characteristics and meat quality of growing lambs.

## Materials and Methods

### Ethical approval

All of the methods used in this experiment have been approved through the Jordan University of Science and Technology (JUST; [Protocol no: 16/03/03/275]) Institutional Animal Care and Use Committee.

### Study period and location

The study was conducted in the Animal Farm at the Agricultural Research and Training Unit at JUST during the period from March to May 2019.

### Animals and experimental procedures

A group of 24 male lambs were randomly selected from a group of 60 lambs that were born on the JUST Animal Farm. Before the study, lambs were weighed, health checked, and treated for internal parasites. At the beginning of the study, there was no difference in the initial body weight (BW) between the two groups; the average BW was 16.9±0.42 kg. Lambs were housed individually in shaded concrete-surfaced pens (1.5×0.75 m). Each pen was equipped with a plastic waterer (7 L) and plastic feeders (10 L). Lambs had *ad libitum* access to feed (110% of the previous day’s intake) and water throughout the experiment. Procedures, nutrient intake, and growth performance data were previously described by Obeidat [6]. In brief, feeding lambs with BCM at 150 g/kg of the dietary dry matter (DM) improved their growth performance and nutrient intake, compared to lambs fed a CON diet.

BCM was obtained from a local oil company (Green Fields Oil Factory, Amman, Jordan) and transported to the JUST campus, where it was sun-dried and ground before being mixed into the diet. Lambs (2-3 months old) were randomly assigned to one of two iso-nitrogenous (160 g/kg CP of dietary DM) treatment diets. Diets were formulated to satisfy the requirements for growing Awassi lambs [9]. Diets were 0 g BCM/kg (BCM0; n=12) or 150 g BCM/kg of dietary DM (BCM 150) to replace part of the barley grain and soybean meal (Table-1). Throughout the study, feed was mixed once every 2 weeks and a sample was taken to analyze its chemical composition. The experiment period was 80 days, of which the first 10 days were used as an adaptation period to the diets, followed by 70 days of data collection. Feed intake was measured daily. Animals were weighed at the beginning of the study and biweekly thereafter.

**Table-1:** Ingredients and chemical composition of diets-containing BCM fed to Awassi lambs.

Item	Diet^1^		

	CON	BCM 150	BCM
Ingredients (g/kg DM)			
Barley grain, whole	500	440	
Soybean meal, 440 g/kg CP (solvent)	200	110	
Black cumin meal	0	150	
Wheat straw	280	280	
Salt	10	10	
Limestone	9	9	
Vitamin-mineral premix 2	1	1	
Feed cost/ton (US$) 3	400	300	
Nutrients (g/kg DM)			
Dry matter	915	924	918
Crude protein	161	163	328
Neutral detergent fiber	293	308	228
Acid detergent fiber	194	198	114
Ether extract	19	34	122
Metabolizable energy, Mcal/kg^4^	2.31	2.70	5.34^5^

1 Diets were: The CON diet or 150 g/kg BCM (BCM 150) of dietary DM.

2 Composition per kg contained (Vitamin A, 600,000 IU; Vitamin D3, 200,000 IU; Vitamin E, 75 mg, Vitamin K3, 200 mg; Vitamin B1, 100 mg; Vitamin B5, 500 mg; lysine 0.5%; DL-methionine, 0.15%; manganese oxide, 4000 mg; ferrous sulfate, 15,000 mg; zinc oxide, 7000; magnesium oxide, 4000 mg; potassium iodide, 80 mg; sodium selenite, 150 mg; copper sulfate, 100 mg; cobalt phosphate, 50 mg, dicalcium phosphate, 10,000 mg.

3 Calculated based on the prices of diet ingredients of the year 2019.

4 Estimated based on tabular values of NRC [9].

5 Adapted from Gokdogan *et al.* [14]. BCM=Black cumin meal, CON=Control, DM=Dry matter

### Slaughter and meat quality measurements

After fasting for 18 h, the lambs were slaughtered at 9:00 h at the Animal Farm facilities of the Agricultural Training and Research Unit at JUST. Immediately before and after slaughter, fasted live, and hot carcass weights were recorded. Immediately after slaughter, non-carcass edible components (i.e., lungs, trachea, heart, liver, spleen, and kidneys) were removed and weighed.

After chilling for 24 h at 4°C, cold carcass weight was recorded to calculate the dressing percentage (cold carcass weight/fasted live weight). Then, linear dimensions (tissue depth [GR], rib fat depth [J], eye muscle width [A], eye muscle depth [B], eye muscle area, and fat depth [C]) were measured on the carcasses and *longissimus* muscles. Carcasses were then cut into four parts (i.e., shoulder, rack, loin, and leg). During cutting, the *longissimus* muscle was excised from the loin cut, immediately vacuum-packed, and stored for 2 weeks at −20 °C for meat quality evaluation. After thawing in a chiller at 4°C, meat quality parameters, measured on *longissimus* muscles, were shear force values, color (CIE L*a*b* coordinates), water holding capacity (WHC), pH, and cooking loss, as described by Obeidat *et al*. [10].

### Statistical analysis

The treatment diet was the only fixed effect. Data were analyzed using a mixed model (PROC MIXED) in SAS (Version 8.1, 2000, SAS Inst. Inc., Cary, NC). When the fixed effects were significant (p≤0.05), least square means were compared using the appropriate pair-wise t-tests.

## Results

The inclusion of BCM did not affect the nutrient content of the feed, except for the ether extract (EE), which was greater in the BCM than the CON diet (Table-1).

The inclusion of BCM increased fasting live weight (p=0.006), hot (p=0.01) and cold (p=0.008) carcass weight, and carcass cut weights (p=0.009) compared with lambs feed the CON diet (Table-2); however, the dressing percentage and non-carcass components did not differ (p≥0.129) between lambs fed the two diets. Fat tail weight tended to be greater (p=0.069) for lambs fed the BCM diet.

**Table-2:** Effects of BCM on carcass, non-carcass components, and dissected leg carcass cut weights and percentages of Awassi lambs.

Item	Diets^1^			

	CON (n=12)	BCM (n=12)	SE	p-value
Fasting live weight (kg)	30.4	34.5	0.85	0.0060
Hot carcass weight (kg)	14.3	16.4	0.48	0.0099
Cold carcass weight (kg)	13.6	15.8	0.50	0.0081
Dressing percentage	44.5	46.0	0.73	0.129
Non-carcass components (kg)^2^	1.43	1.41	0.028	0.5694
Carcass cut weights (kg)^3^	12.0	13.5	0.35	0.0088
Fat tail (kg)	1.58	1.93	0.143	0.0692
Leg weight (kg)	2.220	2.539	0.7061	0.0085
Subcutaneous fat (g /100 g)	12.19	12.84	0.858	0.6049
Intermuscular fat (g /100 g)	1.901	2.030	0.1022	0.2930
Total fat (g /100 g)	14.09	14.87	0.834	0.5235
Total meat (g/ 100 g)	55.79	57.39	1.051	0.3049
Total bone (g /100 g)	22.26	21.35	0.4313	0.1631
Meat to bone ratio	2.51	2.70	0.045	0.0102
Meat to fat ratio	4.15	4.04	0.253	0.7552

1 Diets were: the CON diet or 150 g/kg BCM (BCM 150) of dietary DM.

2 Non-carcass components (Heart, liver, spleen, kidney, and lungs and trachea).

3 Carcass cut (shoulder, racks, loins, and legs). BCM=Black cumin meal, CON=Control, DM=Dry matter

With regards to dissected legs, leg weight (p=0.0085) and the meat to bone ratio (p=0.0102) were higher for lambs fed the BCM diet compared to those fed the CON diet (Table-2); however, subcutaneous fat, intermuscular fat, total fat, total meat, total bone, and the meat to fat ratio were similar (p≥0.1631) for lambs fed the two diets.

Carcassleaner dimension characteristics are shown in Table-3. Compared to lambs fed the CON diet, lambs fed BCM had increased leg fat depth (p=0.005), and eye muscle depth (p=0.045); however, tissue depth (p=0.347), eye muscle area (p=0.68), and fat depth (p=0.221) did not differ between lambs fed the two diets. Rib fat depth (p=0.064), eye muscle depth (p=0.069), and shoulder fat depth (p=0.097) tended to be higher in lambs fed BCM than the CON diet.

**Table-3:** Effects of feeding BCM on carcass leaner dimensions of Awassi lambs.

Item	Diets^1^			

	CON (n=12)	BCM (n=12)	SE	p-value
Leg fat depth (*L3*) (mm)	2.40	3.74	0.271	0.0049
Tissue depth (*GR*) (mm)	9.05	9.46	0.297	0.3473
Rib fat depth (*J*) (mm)	1.85	2.21	0.125	0.0638
Eye muscle width (*A*) (mm)	50.50	51.52	0.394	0.0689
Eye muscle depth (*B*) (mm)	20.15	21.04	0.278	0.0454
Eye muscle area (cm^2^)	8.84	8.95	0.2188	0.6799
Fat depth (*C*) (mm)	1.50	1.73	0.124	0.2212
Shoulder fat depth (*S2*) (mm)	1.30	1.60	0.116	0.0969

1 Diets were: The CON diet or 150 g/kg BCM (BCM 150) of dietary DM. BCM=Black cumin meal, CON=Control, DM=Dry matter, SE=Standard error

Meat quality traits are presented in Table-4. Feeding BCM did not affect meat pH (p=0.180), CL (p=0.781), WHC (p=0.397), shear force (p=0.428), meat whiteness (p=0.864), redness (p=0.475), or yellowness (p=0.166).

**Table-4:** Effects of feeding BCM on meat quality characteristics of Awassi lambs.

Item	Diets^1^			

	CON (n=12)	BCM (n=12)	SE	p-value
pH^2^	5.73	5.74	0.007	0.1803
Cooking loss (g/100 g)	39.1	38.9	0.7	0.7806
Water holding capacity (g/100 g)	26.7	27.9	0.96	0.3970
Shear force (kg/cm^2^)	8.00	8.29	0.320	0.4279
Color coordinates				
L* (whiteness)	37.2	37.1	0.52	0.8641
a* (redness)	3.41	2.95	0.463	0.4745
b* (yellowness)	18.19	18.79	0.285	0.1655

1 Diets were: The CON diet or 150 g/kg BCM (BCM 150) of dietary DM. ^2^pH measured after thawing. BCM=Black cumin meal, CON=Control, DM=Dry matter, SE=Standard error

## Discussion

Using BCM as an alternative feed is reported to have a positive effect on animal growth performance [11], reproduction [12], and health [13]. In the current study, the supplementation of BCM enhanced the carcass characteristics of Awassi lambs. The improvement in carcass characteristics, carcass cuts, and leaner dimensions could be explained by the effects of BCM on average daily gain, nutrient intake, digestibility, and/or N retention in lambs [6]. As shown in Table-1 [9,14], the nutrient content of BCM suggests the importance of using this feed as an alternative source of protein and energy for growing and finishing lambs. The addition of 150 g BCM/kg (on a DM basis) increased the EE content in the BCM 150 diet, compared to the CON diet; this is due to the higher content of EE (122 g/kg) in BCM versus the other ingredients. In other studies, the dietary inclusion of different levels of BCM increased the EE content of the diet [6,15,16]. The contents of other nutrients (CP=328 g/kg, neutral detergent fiber =228 g/kg, acid detergent fiber=114 g/kg) suggest that BCM is an important by-product that could be used to replace soybean meal and barley grains.

Moreover, replacing soybean meal and barley grains with BCM reduced the experimental diet cost by 20%, compared to the CON diet, likely due to the lower cost of BCM compared to the cost of the other feed ingredients. This lower cost highlights the economic benefit of using BCM as an alternative feed for finishing lambs. Similarly, Abdel-Magid *et al*. [3] studied the effect of adding BCM to rations on the economic efficiency of growing calves and reported that rations with BCM had the lowest cost and enhanced calves’ performance, compared to the cost, and effect of a CON diet.

The addition of *N. sativa* seeds significantly improved lambs’ fasting live weight, and hot, and cold carcass weight. Furthermore, BCM significantly improved carcass cut weights, and enhanced fat tail weight. These results are in line with the findings of Cherif *et al*. [7], who reported that the addition of *N. sativa* seeds to a balanced forage to concentrate ration had a significant effect on the hot and cold carcass yields, and tail weights of Barbarine lambs. The positive effect of BCM on carcass traits may be due to the high nutritive components of *Nigella* seeds, containing a mixture of essential fatty acids (e.g., linolenic, oleic, and linoleic acids) that are crucial for body growth and development and cannot be naturally synthesized [17]. These essential fatty acids are a precursor for the essential arachidonic fatty acid, which plays an important role in prostaglandin synthesis [17]. The results of the current study expand on those produced by Obeidat [6], who reported an improvement in the final BW, average daily gain, and feed efficiency of lambs that consumed BCM 150 versus a CON diet. In short, the positive effect of BCM on carcass parameters and leg weight may be explained by the fact that growth performance, energy intake, and digestibility improved in lambs fed the BCM diet compared with those fed a CON diet [6].

In this current study, the improvement of carcass leaner dimension characteristics, such as leg fat and eye muscle depth, was a reflection of the increase in the lambs’ live and carcass weight after the consumption of BCM. Azeem *et al*. [18] reported that an increase in carcass and edible organ percentages may be due to the enhancement of nutrient utilization and protein metabolism in animals consuming BCM-containing diets. Other studies [19-22] support the fact that BCM is an excellent source of protein, which can be supplemented to ruminant diets to improve their live weight, body condition score, and carcass characteristics.

In the current study, meat characteristics were not affected by the inclusion of BCM. Other researchers have reported an impact of BCM on meat quality. Abdallah *et al*. [4] noted an improvement in the meat nutrient content when lambs consumed greater amounts of BMC in their formulated diets. Likewise, Cherif *et al*. [23] observed an improvement in the oxidative and color stability of stored meat from lambs that consumed BCM diets. Furthermore, the meat quality of poultry and rabbits is improved by the addition of BCM to their diets [24,25].

## Conclusion

Feeding growing lambs BCM improved their carcass yield, compared with those fed a CON diet; however, it had no effect on meat quality parameters. Additional research is needed to evaluate the effect of feeding different levels of BCM to sheep or other livestock species.

## Author’s Contributions

BSO: Designed, performed the study, and drafted and revised the manuscript. The author has read and approved the final manuscript.

## References

[1] Department of Statistic (DoS) (2018). Preliminary Report. Agriculture Section.

[2] Zarkawi M (1997). Monitoring the reproduction performance in Awassi ewes using progesterone radioimmunoassay. Small Rumin. Res.

[3] Abdel-Magid S.S, El-Kady R.I, Gad S.M, Awadalla I.M (2007). Using cheap and local non-conventional protein meal (*Nigella sativa*) as least-cost rations formula on performance of crossbred calves. Int. J. Agric. Biol.

[4] Abdallah N.M, Dahal I.M, Abdul Aziz O.A (2012). Effect of adding *Nigella sativa* meal to Awassi lambs growing rations on production and carcass characteristics. Al-Rafedain Agric. J.

[5] Tekeli A (2014). Nutritional value of black cumin (*Nigella sativa*) meal as an alternative protein source in poultry nutrition. J. Anim. Sci. Adv.

[6] Obeidat B.S (2020). The inclusion of black cumin meal improves growth performance of growing Awassi lambs. Vet. Sci.

[7] Cherif M, Ben Salem H, Abidi S (2018a). Effect of the addition of *Nigella sativa* seeds to low or high concentrate diets on intake, digestion, blood metabolites, growth and carcass traits of Barbarine lamb. Small Rumin. Res.

[8] Paarakh P.M (2010). *Nigella sativa* Linn a comprehensive review. Indian J. Nat. Prod. Res.

[9] National Academy of Sciences (2007). Nutrient Requirements of Small Ruminants:Sheep, Goats, Cervids and New World Camelids.

[10] Obeidat B.S, Mahmoud K.Z, Maswadeh J.A, Bsoul E.Y (2016). Effects of feeding *Atriplex halimus* L on growth performance and carcass characteristics of fattening Awassi lambs. Small Rumin. Res.

[11] Ghasemi H.A, Kasani N, Taherpour K (2014). Effects of black cumin seed (*Nigella sativa* L.), a probiotic, a prebiotic and a synbiotic on growth performance, immune response and blood characteristics of male broilers. Livest. Sci.

[12] Zanouny A.I, Abd-El Moty A.K.I, El-Barody M.A.A, Sallam M.T, Abd El-Hakeam A.A (2013). Effect of supplementation with *Nigella sativa* seeds on some blood metabolites and reproductive performance of Ossimi male lambs. Egypt. J. Sheep Goats.

[13] Shokrollahi B, Sharif B (2018). Effect of *Nigella sativa* seeds on growth performance, blood parameters, carcass quality and antibody production in Japanese quails. J. Livest. Sci.

[14] Gokdogan O, Eryilmaz T, Yesilyurt M.K (2015). Determination of energy use efficiency of *Nigella sativa* L. (Black Seed) oil production. Am. Eur. J. Agric. Environ. Sci.

[15] Taha E.A (2017). A study of some wool traits of Barki sheep fed on *Nigella sativa* cake under desert conditions. Alexandria J. Agric. Sci.

[16] Mansour R.S, Nasser A.K, Abo N.Y (2013). The effect of different *Nigella*
*sativa* L seed (cake) concentrations on leukocytes counts and some serum immunological parameters in calves. Tikrit J. Pure Sci.

[17] Datta A.K, Saha A, Bhattacharya A, Mandal A, Paul R, Sengupta S (2012). Black Cumin (*Nigella Sativa* L.) a review. J. Plant Dev. Sci.

[18] Azeem T, Ur-Rehman Z, Umar S, Asif M, Arif M, Rahman A (2014). Effect of *Nigella sativa* on poultry health and production: A review. Sci. Lett.

[19] Hassan S.A, Hassan K.M, Al-Rubeii A (2010). Carcass characteristics of Karadi lambs as affect by different levels of dietary supplement of rumen degradable nitrogen fed with *Nigella sativa*. Afr. J. Biotechnol.

[20] Abd El-Rahman H.H, El-Nomeary Y.A.A, Shoukry M.M, Mohamed M.I (2014). Effect of substitution of cottonseed meal by two various protein sources on productive performance of fattening crossbred calves. Am. Eur. J. Agric. Environ. Sci.

[21] Abd El-Rahman H.H, Abedo A.A, Salman F.M, Mohamed M.I, Shoukry M.M (2011). Partial substitution of cumin seed meal by Jatropha meal as a potential protein source for feed. Afr. J. Biotechnol.

[22] Longato E, Meineri G, Peiretti P.G (2015). Nutritional and zootechnical aspects of *Nigella sativa*: A review. J. Anim. Plant Sci.

[23] Cherif M, Valentic B, Abidi S, Lucianod G, Mattiolid S, Pausellid M, Bouzarraaa I, Prioloc A, Ben Salem H (2018b). Supplementation of *Nigella sativa* seeds to Barbarine lambs raised on low- or high-concentrate diets:Effects on meat fatty acid composition and oxidative stability. Meat Sci.

[24] AL-Beitawi N, El-Ghousein S.S (2008). Effect of feeding different levels of *Nigella sativa* seeds (Black Cumin) on performance, blood constituents and carcass characteristics of broiler chicks. Int. J. Poult. Sci.

[25] AL-Kuhla A.A, Abdullah N.M (2010). The effect of substituting *Nigella sativa* meal as a source of protein in the rations of local rabbits on their productive performance and carcass traits. Iraqi J. Vet. Sci.

